# Antiepileptic drug-loaded and multifunctional iron oxide@silica@gelatin nanoparticles for acid-triggered drug delivery

**DOI:** 10.1038/s41598-024-62248-z

**Published:** 2024-05-18

**Authors:** Nazanin Ghane, Shahla Khalili, Saied Nouri Khorasani, Oisik Das, Seeram Ramakrishna, Rasoul Esmaeely Neisiany

**Affiliations:** 1 Department of Chemical Engineering, Isfahan University of Technology, Isfahan, 84156-83111 Iran; 2 Department of Civil, Environmental and Natural Resources Engineering, Luleå University of Technology, 97187 Luleå, Sweden; 3 Center for Nanotechnology & Sustainability, National University of Singapore, Singapore, 117574 Singapore; 4 Department of Polymer Engineering, Hakim Sabzevari University, Sabzevar, 9617976487 Iran; 5 Biotechnology Centre, Silesian University of Technology, Krzywoustego 8, 44-100 Gliwice, Poland

**Keywords:** Phenytoin, Superparamagnetic nanoparticles, Silica, Gelatin, Antiepileptic drug, pH-sensitivity, Drug delivery, Engineering, Materials science

## Abstract

The current study developed an innovative design for the production of smart multifunctional core-double shell superparamagnetic nanoparticles (NPs) with a focus on the development of a pH-responsive drug delivery system tailored for the controlled release of Phenytoin, accompanied by real-time monitoring capabilities. In this regard, the ultra-small superparamagnetic iron oxide@silica NPs (IO@Si MNPs) were synthesized and then coated with a layer of gelatin containing Phenytoin as an antiepileptic drug. The precise saturation magnetization value for the resultant NPs was established at 26 emu g^-1^. The polymeric shell showed a pH-sensitive behavior with the capacity to regulate the release of encapsulated drug under neutral pH conditions, simultaneously, releasing more amount of the drug in a simulated tumorous-epileptic acidic condition. The NPs showed an average size of 41.04 nm, which is in the desired size range facilitating entry through the blood–brain barrier. The values of drug loading and encapsulation efficiency were determined to be 2.01 and 10.05%, respectively. Moreover, kinetic studies revealed a Fickian diffusion process of Phenytoin release, and diffusional exponent values based on the Korsmeyer-Peppas equation were achieved at pH 7.4 and pH 6.3. The synthesized NPs did not show any cytotoxicity. Consequently, this new design offers a faster release of PHT at the site of a tumor in response to a change in pH, which is essential to prevent epileptic attacks.

## Introduction

Epilepsy, a persistent neurological condition, arises due to excessive abnormal neuronal activity and sudden discharge of brain neurons^1^. Among individuals diagnosed with brain metastases, symptomatic epilepsy typically arises as a result of the brain lesion in most cases^2,3^. The pathophysiological mechanisms of triggering epileptic seizures in these cases are due to disrupting the neuronal connections, inhibiting the local network regulation, dysfunction of the BBB, resulting in heightened vascular permeability, causing necrosis and hemosiderin deposition, peritumoral edema, and inflammation^4^. Even in the absence of seizures, most primary or metastatic brain tumor cases are prophylactically administered tumor-associatednts, because epilepsy may occur later during the disease^5^.

Phenytoin (PHT), a common drug to prevent epilepsy in patients with cerebral metastases, is a barbiturate derivative^6^. The presence of two phenyl groups at the C5 position of the hydantoin is accountable for its anticonvulsant properties^7,8^. The mechanism by which PHT operates involves the inhibition and obstruction of ion channels within cells of the central nervous system (CNS). However, the high-dose use of PHT to achieve a therapeutic plasma concentration range can lead to various side effects. These side effects encompass liver disease, reduced bone mineral content, megaloblastic anemia, diminished serum folate levels, and gingival enlargement^9^ while, during seizures, the intracellular pH value decreased^10^.

The concept of theranostics has been offered to overcome various limitations in drug delivery systems by concurrently combining monitoring and treatment of diseases^11,12^. Advancements in nanotechnology have enabled the creation of nanostructures, both two-dimensional (2D) and 3D, that incorporate diverse biomaterials^13–17^. Consequently, the NPs, having a size range of 1–100 nm represent a promising approach to address the systemic toxicity and limitations around drug delivery across the BBB^18^. The developed NP carriers exhibit a considerable capability for both drug encapsulation and safeguarding drugs from degradation. Additionally, they can be absorbed by Kupffer cells in the liver when introduced into the body’s systemic circulation^19^. To achieve this system, a core–shell structure can be used to gather a broad variety of materials such as drugs, metal oxides, and polymers^11^. In this context, the magnetic core component offers notable capabilities for theranostic purposes, such as enhancing contrast in magnetic resonance imaging (MRI), achieving a multimodality treatment due to hyperthermia properties, and targeted drug delivery by guiding the NPs to a specific site via an external magnetic field (MF)^20^. In this context, iron oxide nanoparticles (IONPs) reduce the intensity of MRI signals on T_2_ and T_2_*-weighted images, which causes a MF inhomogeneity^21^. Su et al. developed an iron oxide@silica-drug/graphene quantum dot nanoprobe for fluorescence/MRI imaging and fluorescence resonance energy transfer-based drug release (DR) sensing^22^. They applied a thermal decomposition method to synthesize the magnetic core part of nanoprobes. The researchers examined how the concentration of the superparamagnetic core impacts T_2_-weighted signal intensity. Their findings revealed a direct correlation between relaxation time and the concentration of IO, which reveals the development of an efficient T_2_-weighted MRI contrast agent. Moreover, gelatin-coated NPs, as acid-responsive NPs, can effectively control the release of antiepileptic drugs at the target region in patients with brain tumors, where rapid cell growth increases the metabolic rate and causes acidosis within and around the tumor mass^23^.

**Figure 1:**
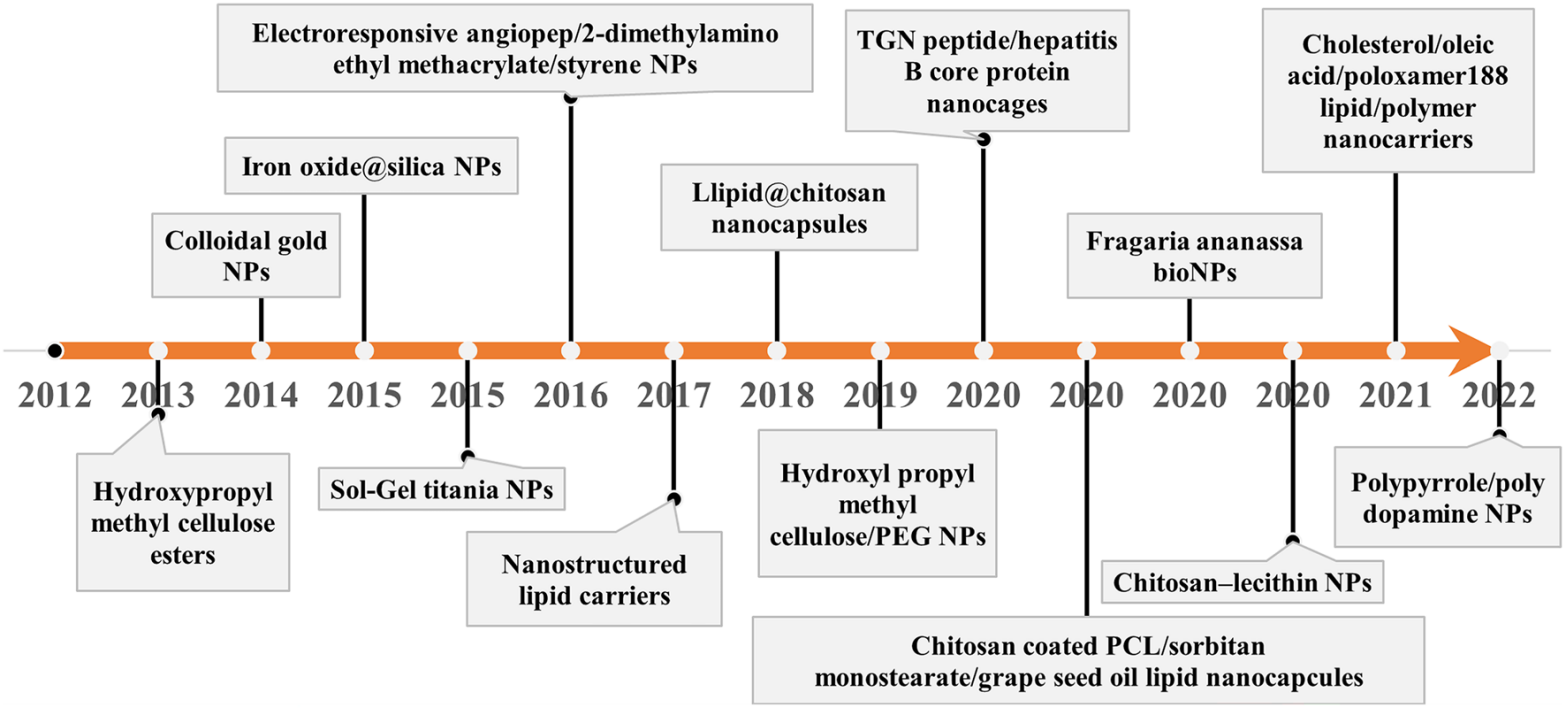
Timeline of milestones in PHT-loaded NPs for epileptic seizures over the last ten years, based on the results from references^24–26,28–38^.

Antiepileptic drugs have been encapsulated in several nanocarriers including sol–gel titania NPs, silica NPs, poly(lactic-co-glycolic acid) (PLGA)-chitosan NPs for intranasal delivery, PLGA-polyethylene glycol (PEG) NPs, poly(ε-caprolactone)-chitosan nanocapsules, lipid nanocarriers, and hydroxypropyl methylcellulose-PEG carriers^18,24^. In another research,^25^ developed an electroresponsive hydrogel nanocarrier containing PHT and reported that their NPs have a high distribution and electroresponsibility in the brain, which causes a strong release of drugs during seizures. Rosillo-de la Torre et al.^26^ synthesized iron oxide@silica magnetic NPs (IO@Si MNPs) and loaded PHT in silica by an adsorption process. Their final product had a mean diameter of ca. 24 nm and released 94% of its drug during the first 4 h of the test. Liu, Yang, and Ho^27^ loaded the antiepileptic drug carbamazepine in carboxymethyl chitosan NPs for an intranasal administration; the particle size of carriers was 219 nm, and DL and entrapment efficiency were 35 and 80%, respectively. PHT-loaded nano-sized particles for epileptic seizures that have been reported in the literature are summarized in Fig. 1.

In the present work, a new design of drug-loaded and core-double shell superparamagnetic NPs was developed and their efficacy as an anti-epileptic drug carrier was investigated. In this new design, drug-loaded and core-double shell structure of superparamagnetic NPs, the drug is loaded in the outer polymeric shell, and not in the silica part. Consequently, a change in pH condition leads to faster PHT release at the site of the tumor, which is essential to prevent epileptic attack. Firstly, iron oxide magnetic nanoparticles (IO MNPs), having a size of 28 nm, were developed through a modified co-precipitation technique, followed by the application of a silica shell coating. In the following step, NPs were exposed to a polymeric solution containing PHT to create a PHT-loaded Gel layer around the surface of NPs as the second shell. Finally, in vitro studies and biocompatibility tests were conducted and their effectiveness towards DL and pH responsibility was investigated. In vitro, release kinetics was according to the Parabolic Diffusion model.

## Methods

### Chemical and reagents

Iron (III) chloride hexahydrate (FeCl_3_•6H_2_O), Iron (II) chloride tetrahydrate (FeCl_2_•4H_2_O), ethanol, oleic acid, 3-(4,5-dimethylthiazol-2-yl)-2,5-diphenyltetrazolium bromide (MTT), penicillin–streptomycin, phosphate-buffered saline (PBS), dimethyl sulfoxide (DMSO), and cetyltrimethylammonium bromide (CTAB) were all provided by Sigma-Aldrich Company (Germany). Ammonium hydroxide, ammonium nitrate (NH_4_NO_3_), chloroform, tetraethyl orthosilicate (TEOS), and ethyl acetate were provided by Merck Company in Germany. Phenytoin (PHT), an antiepileptic drug, was acquired from Darupakhsh in Iran. Gelatin (Type A) (Gel) was provided by Fluka in Switzerland. The culture medium, which included fetal bovine serum (FBS), was purchased from Gibco Company in the USA. All materials were used in their as-received condition without further processing.

### Core-double shell MNP synthesis

#### Synthesis of MNPs

To synthesize the MNPs, an adapted co-precipitation technique using iron (II) and iron (III) chlorides in a molar ratio of 1:2 was employed. Initially, they were dissolved in 100 mL of deionized (DI) water in a three-neck flask, with vigorous stirring facilitated by a mechanical stirrer (Overhead laboratory stirrer SH-II-7C, Huanghua Faithful Instrument Co., China). This process was conducted under a nitrogen (N_2_) gas atmosphere at 80 °C. Subsequently, 50 mL of 25% ammonium hydroxide was swiftly introduced into the solution, resulting in an immediate color change to black. After 30 min had elapsed, oleic acid was introduced to the mixture and it was allowed to complete the reaction at 80 °C for 1.5 h under N_2_. Finally, the collected products were magnetically separated, followed by centrifuging (High-Speed Centrifuge, CFG-16D, Infitek Co., China), washing with DI water, and placing in chloroform^39^.

#### Preparation of silica-coated MNPs

Oleic acid-stabilized NPs were coated with silica. Following this procedure, oleic acid-coated NPs (6.0 mg Fe/mL) were introduced into a 5 mL aqueous CTAB solution and vigorously stirred at 50 °C for half an hour. Subsequently, it was stirred for an additional 10 min at 70 °C to allow for the evaporation of chloroform, resulting in the formation of a clear, black dispersion^39^. Following this step, the obtained dispersion was augmented with the addition of 45 mL of Millipore water, 3 mL of ethyl acetate, 0.5 mL of TEOS, and 0.3 mL of NaOH solution. The pH was set at 12 by incorporating an aqueous NaOH solution^40^. Subsequently, the stirring of the mixture was continued at 70 °C with refluxing for 3 h. Lastly, the NPs were harvested through high-speed centrifugation, yielding a brown precipitate. This precipitate underwent a thorough washing process with ethanol and DI water, carried out thrice to eliminate any unreacted reagents. Subsequently, the resultant NPs were dried in a vacuum oven (Drying oven VD 23, BINDER GmbH, Germany) at room temperature^41–44^. To eliminate CTAB, the prepared NPs were dispersed in ethanol containing NH_4_NO_3_ for 6 h. The procedure of ion exchange was repeated 3 times. The NPs were subsequently retrieved through magnetic separation, followed by centrifugation, and underwent several rounds of washing with DI water and ethanol. Finally, they were subjected to vacuum drying.

#### Preparation of PHT-loaded *IO*@Si@Gel MNPs

A 1% (w/v) solution of Gel polymer was prepared as the shell solution for drug encapsulation. PHT was introduced into the solution while the ratio of the drug to polymer was set at 20 wt%. Next, 50 mg of IO@Si MNPs were gently agitated with the drug-loaded shell solution at 50 °C for a period of 6 h. Subsequently, cold DI water was rapidly added to the mixture at 4 °C. The resulting MNPs were subjected to centrifugation, washed with DI water, redispersed three times, and finally subjected to freeze-drying^45^.

### Characterizations

#### Chemical study

The chemical characteristics and functional groups of all specimens including IO MNPs, IO@Si MNPs, and IO@Si@Gel MNPs were determined by Fourier-transform infrared (FTIR, resolution = 4, scan times = 32, Rayleigh WQF-510A, China) spectroscopy using KBr pellets.

#### Crystalline structures

The crystallinity and ordered structures of the IO MNPs and IO@Si MNPs powder were investigated using X-ray diffraction (XRD) at λ = 1.54 Å on (Cu Kα radiation, PMD Philips X-Pert, Philips, Netherland). In this regard, the powder samples were dried at first and then placed onto an amorphous glass plate and XRD spectra were measured in the 2θ range of 10–99.

#### Specific surface areas

The specific surface areas of IO@Si MNPs were assessed using the Brunauer–Emmett–Teller (BET) technique, employing a Nano SORD instrument in a linear relative pressure.

#### Thermal properties

The thermal characteristics of IO MNPs, IO@Si MNPs, and IO@Si@Gel MNPs were evaluated by differential scanning calorimetry (DSC) with a 350 mW instrument from BÄHR-Thermoanalyse GmbH in Germany. The employed heating rate was adjusted at 10 °C per minute.

#### Superparamagnetic properties

The superparamagnetic properties and hysteresis curve of the IO MNPs and IO@Si@Gel MNPs were analyzed by a vibrating sample magnetometer (VSM) on a (MDKB, Magnetic Daghigh Kavir, Iran).

#### Morphological study

All samples, including IO MNPs, IO@Si MNPs, and PHT-loaded IO@Si@Gel MNPs, underwent a gold sputter-coating process lasting 10 min. Subsequently, they were subjected to morphological studies utilizing a scanning electron microscopy (SEM), on a Philips XL30 instrument from Eindhoven, Netherlands. Moreover, during morphological studies by SEM, an energy-dispersive X-ray analysis (EDX) spectrum was conducted.

For further morphological investigation by transmission electron microscopy (TEM), the IO MNPs and PHT-loaded IO@Si@Gel MNPs were dispersed in ethanol dropped on the carbon-coated copper grids. The MNPs’ average size and distribution were measured by ImageJ software.

#### Hydrodynamic particle size and surface charge

To evaluate the hydrodynamic particle size and Zeta potential of the IO MNPs, IO@Si MNPs, and PHT-loaded IO@Si@Gel MNPs, the dynamic light scattering (DLS-Zeta, HORIBA, Ltd., Japan) at room temperature was employed, while the NPs were suspended in DI water. Before measurement, each sample solution was sonicated (SONICA ultrasonic bath, ultrasonic cleaners, Italy) for 10 min to ensure a uniform dispersion.

#### DL and EE of the PHT in MNPs

The DL and EE of PHT within the PHT-loaded IO@Si@Gel MNPs were assessed using UV–vis spectroscopy (Rayleigh, China). The measurements were conducted at a specific wavelength of 213 nm. Before this analysis, a calibration curve was established, covering the concentration range of 10–13.64 ppm of PHT in PBS. Then, these values were calculated as the folowing:

1 $${\text{DL}}\left( {{\text{wt}}\% } \right) = \left( {{\text{Weight of loaded PHT}}/{\text{Weight of final NPs}}} \right) \times 100$$

2 $${\text{EE}}\left( {{\text{wt}}\% } \right) = \left( {{\text{Weight of loaded PHT}}/{\text{Weight of feeding PHT}}} \right) \times 100$$

#### In vitro studies

##### In vitro DR study

For the assessment of the DR release, in vitro tests were carried out^46^, as such, PHT-loaded IO@Si@Gel MNPs, 5 mg of NPs were dispersed in PBS, placed in 10 KDa dialysis bags, and then soaked in PBS solution at two pH values (7.4 and 6.3) and 37 °C under gentle shaking. At regular intervals, the solutions (4 ml) were collected and evaluated to determine the quantity of PHT released using a UV–vis spectrophotometer at the absorbance of 213 nm.

##### Kinetics analysis of PHT release

Kinetic analysis of PHT release involved fitting the in vitro release data to several models, which encompassed the zero and first order, Korsmeyer-Peppas, Higuchi, and Parabolic Diffusion models. The aforementioned models were utilized to elucidate both the kinetics and release mechanism of PHT from NPs^47^. The correlation coefficient (R^2^) of these models was employed to determine the most suitable fitting model.

##### Biocompatibility evaluation

In this research, L-929 cells (NCTC clone 929: CCL 1, American Type Culture Collection) were cultivated in a medium containing 10% FBS and 1% penicillin–streptomycin. They were cultured under controlled conditions in a humid environment with 5% CO2 at 37 °C. To assess in vitro cell activity, the MTT assay was conducted on days 1, 2, and 3 of incubation at 37 °C, within an environment comprising 5% CO_2_ and 90% relative humidity. During the assay, MTT solution was introduced, and the samples were allowed to incubate at 37 °C for three hours. Following this, DMSO was added to each well to dissolve the purple formazan crystals. The absorbance of each specimen was subsequently recorded at 570 nm on a spectrophotometer (Hyperion MPR4, Germany). As a reference, a tissue culture plate was utilized, following ISO 10,993-5 guidelines^21^. The percentage of cell viability is determined as follows:

3 $$\% {\text{Cell}}\;{\text{viability}} = \frac{{{\text{I}}\;{\text{sample}} - {\text{I}}\;{\text{DMSO}}}}{{{\text{I}}\;{\text{control}}}} \times 100$$

In this equation, “I_sample_” represents the fluorescence intensity of each individual sample, “I_DMSO_” stands for the fluorescence intensity of DMSO, and “I_control_” denotes the fluorescence intensity of the cells incubated solely with the culture medium.

#### Statistical analysis

All the experiments were conducted in triplicate, and the results were presented as average values accompanied by standard deviations. The statistical comparisons between different groups were performed by one-way analysis of variance (ANOVA). The obtained *p*-values less than 0.05 were considered statistically significant.

## Results and discussion

### Core-double shell MNP design and synthesis

**Figure 2:**
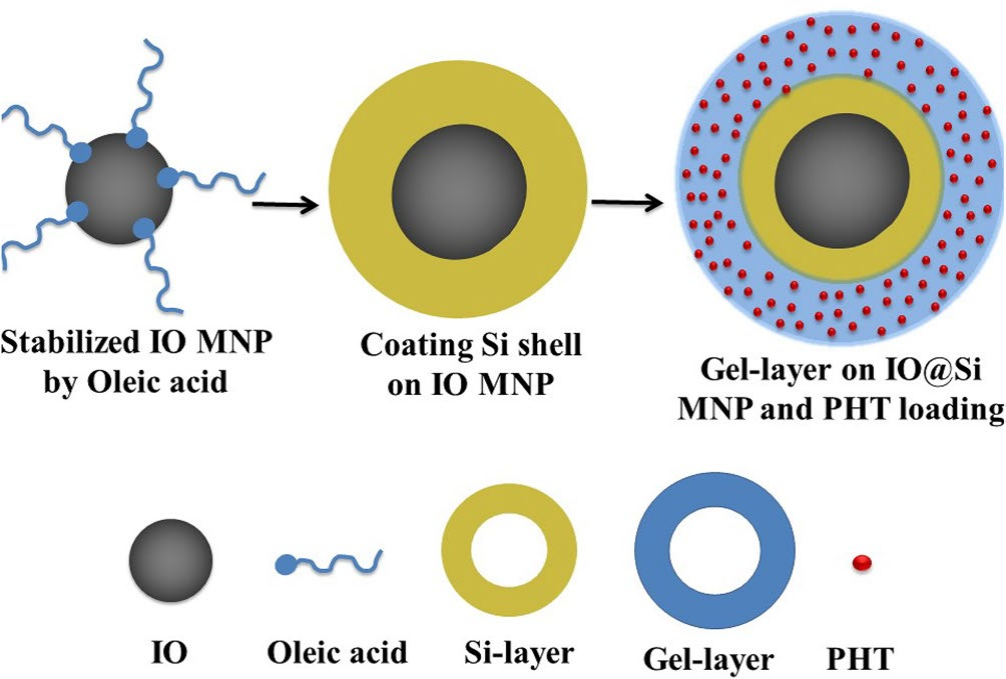
Representative illustration of the core-double shell arrangement of PHT-loaded IO@Si@Gel MNPs.

In this study, brain-targeting PHT-loaded core-double shell structured NPs were synthesized in accordance with the procedures demonstrated in Fig. 2. First and foremost, the hydrophobic oleic IO MNPs, as the core, were synthesized based on a modified co-precipitation method. Subsequently, a silica layer was applied as a shell to envelop the surface of IO MNPs, yielding IO@Si MNPs. This was achieved using the conventional Stober method, wherein CTAB served as a stabilizing agent during the transition of hydrophobic IO MNPs to an aqueous medium. Finally, a PHT-loaded polymer shell was fabricated around IO@Si MNPs to prepare the PHT-loaded IO@Si@Gel MNPs. The magnetization of the core part creates a unique opportunity to provide a targeting capability for these NPs. PHT-loaded IO@Si@Gel MNPs can be directed toward particular or pathological spots by assisting an external MF, thereby enhancing the precision of drug-loaded NP targeting^48^. Moreover, adding this hydrophilic gel layer plays a crucial part in improving the circulation time and the distribution in the body along with transporting the PHT and releasing the drug during its biodegradation. Without an effective surface coating, MNPs tend to precipitate in the physiological environment^49^. NPs treated with oleic acid had an appropriate suspension stability in chloroform even after 1 day of dispersion. Additionally, the final PHT-loaded IO@Si@Gel MNPs had good suspension stability after 7 h in DI water.

### Characterizations

#### Physicochemical properties analysis

**Figure 3:**
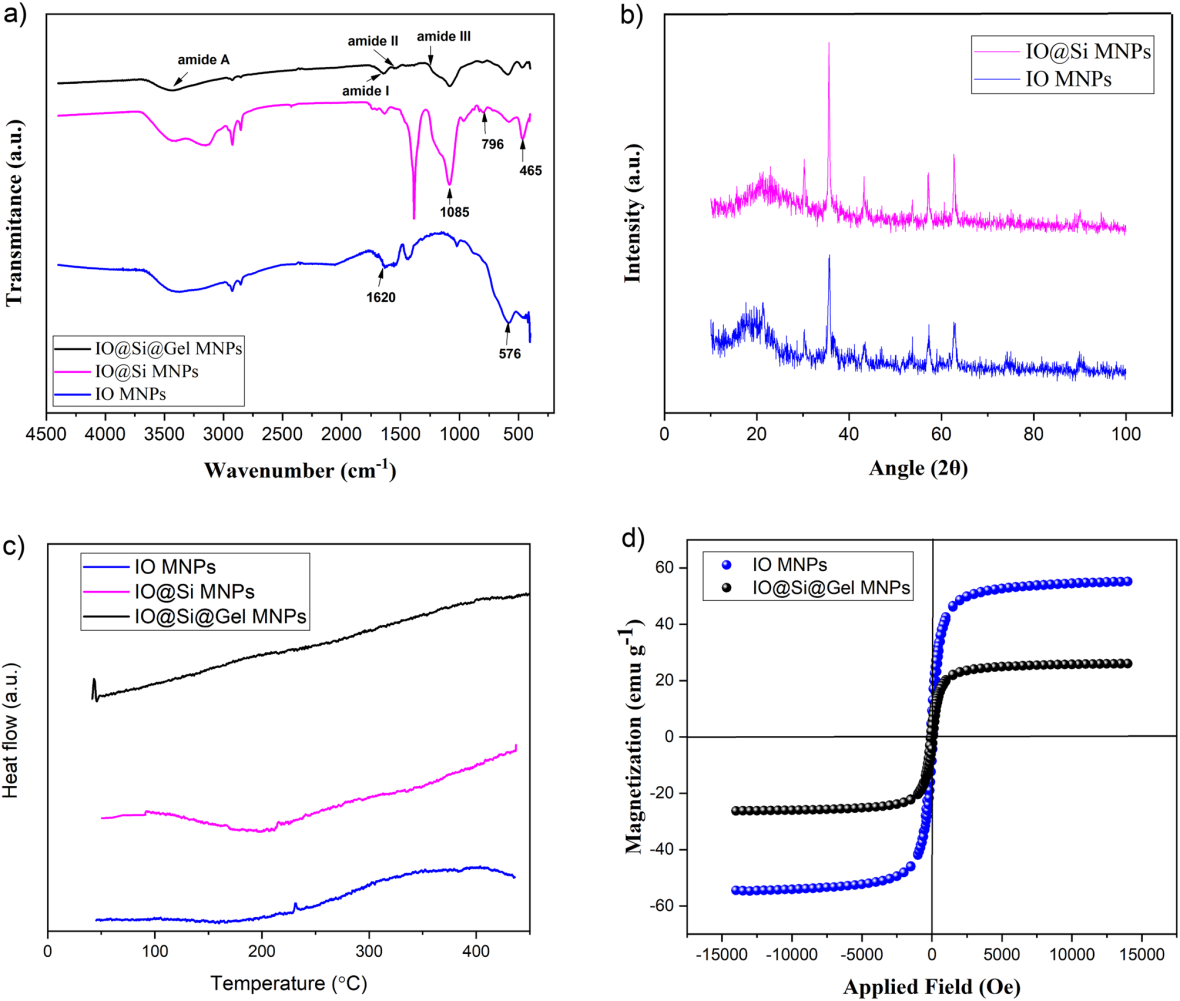
The physicochemical properties; (**a**) The FTIR spectra of IO MNPs, IO@Si MNPs, and IO@Si@Gel MNPs; (**b**) XRD pattern of IO MNPs, and IO@ Si MNPs; (**c**) DSC thermograms of IO MNPs, IO@Si MNPs, and IO@Si@Gel MNPs; d) VSM curve of IO MNPs and IO@Si@Gel MNPs.

To chemically explore the IO-core, Si-shell, and Gel-shell structures, FTIR was conducted. As presented in Fig. 3a, the absorption bands that appeared at 576 and 1620 cm^−1^ correspond to the stretching vibrations of Fe–O bonds and the physical absorption of H_2_O on IO MNPs, respectively. Additionally, a broad peak spanning from 3000 to 3620 cm^−1^ can be associated with the stretching vibration of O–H bonds originating from Fe–OH or H_2_O on the surface of IO MNPs^50^. The silica shell on the surface of IO-core MNPs was confirmed by FTIR, with characteristic absorption band at 1085 and 796 cm^−1^ ascribing to Si–O stretching vibrations, and a peak at 465 cm^−1^ attributed to the Si–O bending vibration, signifying the silica shell formation^51^. Furthermore, the final surface coating of MNPs with a gel shell as validated in Fig. 3a, is evidenced by the specific absorption bands at 1645 , 1539, 1240, and 3436 cm^−1^, which are associated with the functional groups of amide I, II, III, and amide A, respectively^47^.

The crystal structures of the synthesized IO MNPs and IO@ Si MNPs powder were examined through XRD tests, and the resulting patterns are displayed in Fig. 3b. The diffraction peaks observed at 2θ of 30.4°, 35.9°, 43.3°, 53.6°, 57.4°, and 63° are ascribed to the (220), (311), (400), (422), (511), and (440) planes, respectively. These patterns correspond to the cubic phase of pure Fe3O4, featuring a face-centered cubic structure in accordance with the data found in the Joint Committee on Powder Diffraction Standards (JCPDS) card (No. 88–315, α = 8.375)^52^. When the MNPs were coated by silica, a strong and broad peak around 20° corresponding to the silica phase appeared. The XRD patterns indicated the generation of magnetite Fe_3_O_4_ NPs. The BET surface area for NPs after coating a layer of silica (IO@Si MNPs) was also measured and found to be 46 m^2^ g^−1^. The silica layer can provide a surface for coating the next layer containing drugs and gelatin. XRD was applied to confirm the formation of a silica shell on the surface of magnetite Fe_3_O_4_ in Iron oxide NPs. In other words, XRD was performed only to indicate the generation and the successful formation of an amorphous phase of silica at the surface of NPs.

Thermal properties, such as the phase change temperature and enthalpies in the nanocarrier were revealed in the DSC results. Figure 3c shows the DSC thermograms of IO MNPs, IO@Si MNPs, and IO@Si@Gel MNPs. According to these results, IO MNPs had a broad endothermal peak of around 163 °C, which corresponds to the vaporization of oleic acid on the surface of IO MNPs^53^. Moreover, a slight endothermic transition at around 383 °C corroborates the crystallization of the magnetite phase^54^. In the case of IO@Si MNPs, as shown in Fig. 3c, there is no significant peak for the silica, which indicates the successful removal of the CTAB from the structure.

Retention of magnetic properties and achieving a high magnetization value are the most important aspects of ensuring an efficient MRI. The magnetic nature of IO MNPs and IO@Si@Gel MNPs were analyzed by VSM measurements, as presented in Fig. 3d. All hysteresis loops are S-like curves with negligible remanence and coercivity at room temperature, which indicated the superparamagnetic nature of all samples. The specific saturation magnetization value (Ms) for pristine IO MNPs was 55 emu g^−1^. The Ms value for IO@Si@Gel MNPs was 26 emu g^−1^, which is sufficient for the separation of composites with a magnet. The successive silica and Gel coating on the surface of IO MNPs reduced the Ms value. These results showed that despite coating, the inherent magnetic property was retained and made the final NPs as good MRI contrast agents. These MNPs can potentially be magnetically targeted by guiding them to particular regions^55^.

**Figure 4:**
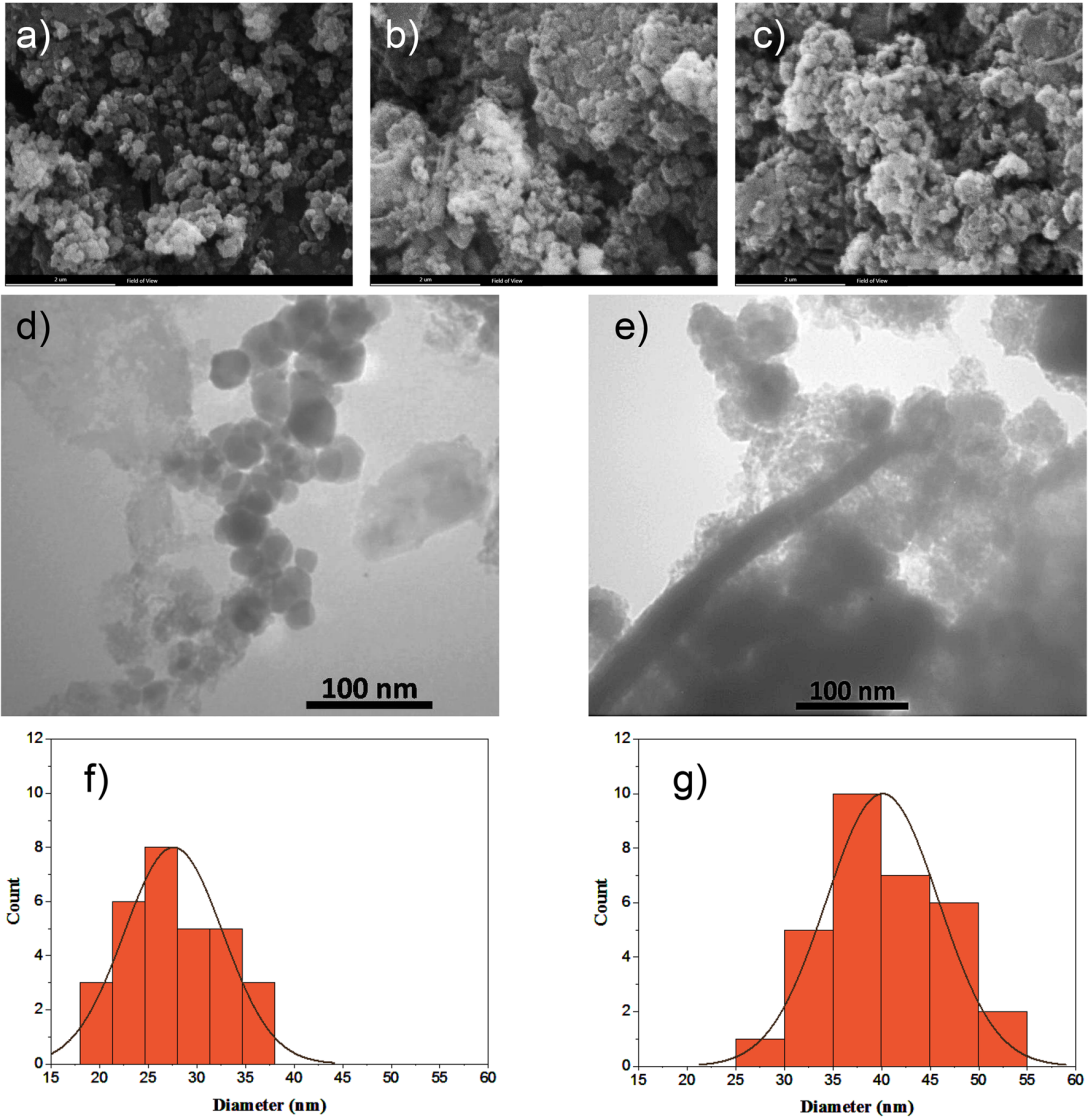
The SEM images of (**a**) IO MNPs, (**b**) IO@Si MNPs, and (**c**) PHT-loaded IO@Si@Gel MNP; TEM images of (**d**) IO MNPs and (**e**) PHT-loaded IO@Si@Gel MNP; size distributions of (**f**) IO MNPs and (**g**) PHT-loaded IO@Si@Gel MNP.

The parameters of the particle size and surface charge primarily impact the fate of NPs after injection. These parameters determine the recognition of NPs or the lack thereof by the defense mechanism of the body^19^. Hence, SEM and TEM images were generated to elucidate the NPs’ morphology, size, and distributions (Fig. 4). The IO MNPs exhibited an almost spherical shape characterized by a smooth surface, having average particle size of 28 nm (Standard Deviation (SD) = 6.1), as shown in Fig. 3. In the case of PHT-loaded IO@Si@Gel MNPs, almost a spherical shape was achieved. These final NPs showed an average particle size of 41 nm (SD = 6.35), which is expected to increase the circulation time in blood, as well as enhance the site-specific targeting significantly. The TEM images showed the structure of the NPs contained an outer bright silica/Gel halo in the case of PHT-loaded IO@Si@Gel MNPs^56^. The ultra-small MNPs have the potential to penetrate the BBB themselves and access the tumor microenvironment. Moreover, focused ultrasound can deliver (monitored by a MRI) them to the CNS and disrupt the BBB^55^. In addition to their improved permeability, magnetite cores within this particular diameter range possess inherent magnetic moments. Consequently, they can be activated by an external and alternating MF to produce ample heat by relaxation mechanisms within the tumor region, enabling effective hyperthermia-based thermotherapy^57^. The microvessels also increase the possibility of accumulating these ultra-small MNPs only in the tumor vessels^58^. In Fig. 4e, there are some agglomerated NPs. In another study by Ebadi et al.,^59^ this occurrence was attributed to the dipole–dipole interaction, free surface energy, and magnetic property of the NPs. This study synthesized Fe_3_O_4_ NPs coated by polyvinyl alcohol/Zn/Al-layered double hydroxide/drug and reported a size distribution of 30 nm and 95 nm for Fe_3_O_4_ NPs and final NPs, respectively, while the present study achieved a remarkable reduction in final NPs’ diameter.

**Figure 5:**
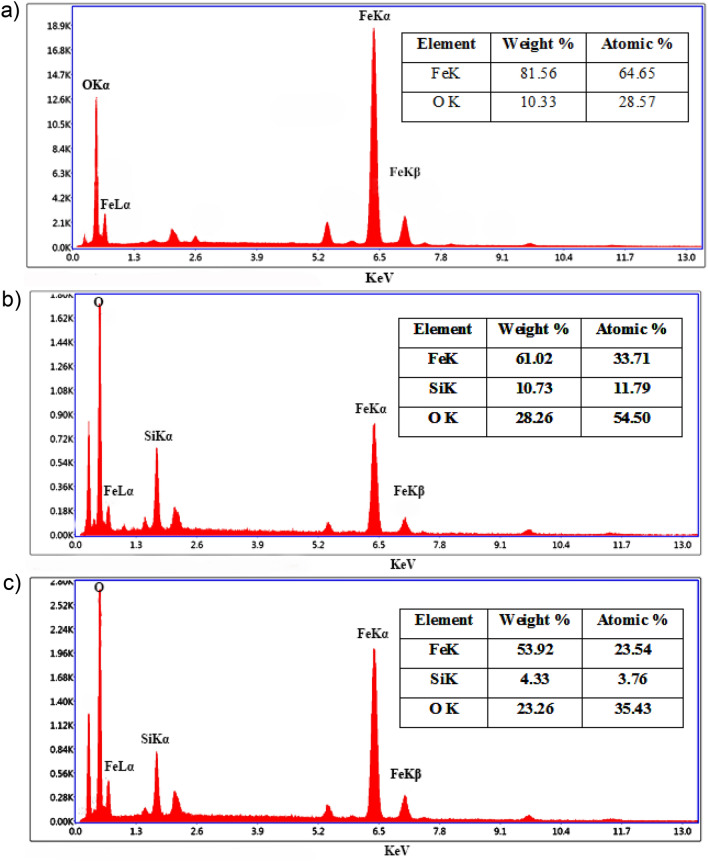
EDX results of (**a**) IO MNPs, (**b**) IO@Si MNPs, and (**c**) IO@Si@Gel MNPs.

As shown in Fig. 5, the EDX patterns illustrated the presence of Fe atoms in all the samples, with a strong and sharp peak in IO MNPs. By adding the silica shell, the percentage of Fe decreased and the EDX spectra showed a strong peak corresponding to Si atoms, which reveals that the IO MNPs were successfully coated by silica (Fig. 5b). After coating NPs with Gel (Fig. 5c), a significant decrease in the percentage of Fe and Si occurred due to the polymeric Gel layer formed on the surface of NPs. Furthermore, there was no overlap in the peaks, which indicates that the nanostructured support materials were free of unreacted precursors or contaminants during synthesizing^28^.

The investigation of the NPs’ morphological characteristics aimed to establish the fundamental physicochemical prerequisites necessary for bypassing the BBB. Based on DLS findings, as presented in Table 1, the hydrodynamic diameter of the IO MNPs measured 117 nm. It is because of the fact that the ultra-small IO MNPs tend to aggregate in the aqueous solution due to their size and because of the oleic acid, with its hydrophobic nature, on the surface of NPs. However, by adding a hydrophilic modification surface, such as silica, the stability and as a result the cell viability of internalized NPs increase, which prolongs their presence within the cells and enhances the efficiency of these contrast agents for long-term tracking^56^. After coating SiO_2_ onto the IO MNP surface, the diameter changed to 72 nm.

**Table 1:** DLS and Zeta potential results of the NPs.

Sample	Hydrodynamic diameter (nm)/Polydispersity index	Zeta potential (mV)
IO MNPs	117/0.8	− 14.3
IO@Si MNPs	72/0.5	− 34
IO@Si@Gel MNPs	89/0.6	− 19.2
PHT-loaded IO@Si@Gel MNPs	81/0.6	− 17.5

The observed diameter measurement was greater than what was seen in SEM images. This discrepancy can be attributed to the hydration layer formed on the IO@Si MNPs’ surface, stemming from robust intermolecular interactions within the Si–O bonds present in the aqueous solution^58^. These small hydrodynamic sizes are promising to circumvent the reticuloendothelial system, causing more maintenance in the blood circulatory system, and potentially passing through the BBB around the brain tumor region^60^. Coating the second layer of the shell increased the IO@Si@Gel MNPs and PHT-loaded IO@Si@Gel MNPs sizes to 89 nm and 81 nm, respectively. A small change occurred in the size of MNPs after loading the PHT and the same has been observed in previous research^24^.

The zeta potential, being an electrostatic potential, is contingent on both the surface charge and the NP environment. This can undergo alterations during surface modification or functionalization following the coating process^48^. The amount of zeta potential is one essential factor that determines the fate of NPs in the human body. According to the Zeta-potential assay, as shown in Table 1, the IO MNPs with a surface coating of oleic acid had a negative value of − 14.3 mV. The IO MNPs exhibit a substantial surface-to-volume ratio, which corresponds to high surface energies. Therefore, they exhibit a tendency to aggregate, a behavior aimed at minimizing their surface energies. In addition, naked IO NPs display heightened chemical reactivity and are susceptible to oxidation when exposed to air. Hence, it is crucial to apply an appropriate surface coating to ensure the stability of these NPs. In this study, oleic acid was utilized during the preparation process to passivate the surface of the NPs, forming a protective monolayer that enhances stability^60,61^. Following the silica layer deposition onto the surface of the IO MNPs, the IO@Si MNPs’ surface charge was elevated to − 34 mV, primarily attributable to the negative charge owing to the hydroxyl groups present in the silica. After coating the last layer of Gel, the surface charges of IO@Si@Gel MNPs were changed to − 19.2 mV, confirming the silanol surface re-exposure, which revealed that the last shell of polymer is formed successfully^45^. These results point to the occurrence of layer-by-layer coating to generate the IO@Si@Gel MNPs. The value of the zeta potential of PHT-loaded IO@Si@Gel MNPs changed from − 19.2 to − 17.5 mV, possibly through loading PHT as a weakly acidic drug.

#### DL and EE of PHT in MNPs

There are several challenges to encapsulating the high amount of drugs for controlled drug delivery in polymeric NPs via traditional methods^62^. Since the small NPs tend to decrease their total stability, causing an undesired burst release effect decreasing their efficacy whereas the large ones show slower in vitro release profiles. Moreover, a systematic delivery reduces their efficacy due to circulating clearance. Therefore, the utilization of a core-shell morphology featuring a magnetically controllable core along with a hybrid shell can effectively address the previously mentioned challenges^62^. To investigate the DL capacity of the IO@Si@Gel MNPs consisting of a polymeric shell, the DL and EE of NPs were determined. The DL was determined to be 2.01%, which was significantly increased for the PHT as an anti-epileptic drug, compared to the results reported previously^26^. In a study, Torre et al. loaded PHT into the silica shell of IO@Si MNPs and reported a percentage of 0.25 as the DL % value, while the current study provides a more efficient approach for increasing the PHT loading in MNPs^26^. Moreover, the EE % for PHT-loaded IO@Si@Gel MNPs was calculated to be 10.05%, indicating that PHT was encapsulated in the NPs despite a thin layer of the Gel shell in the structure of NPs. These results suggest that the IO@Si@Gel MNPs could be an effective PHT carrier.

#### In vitro studies

**Figure 6:**
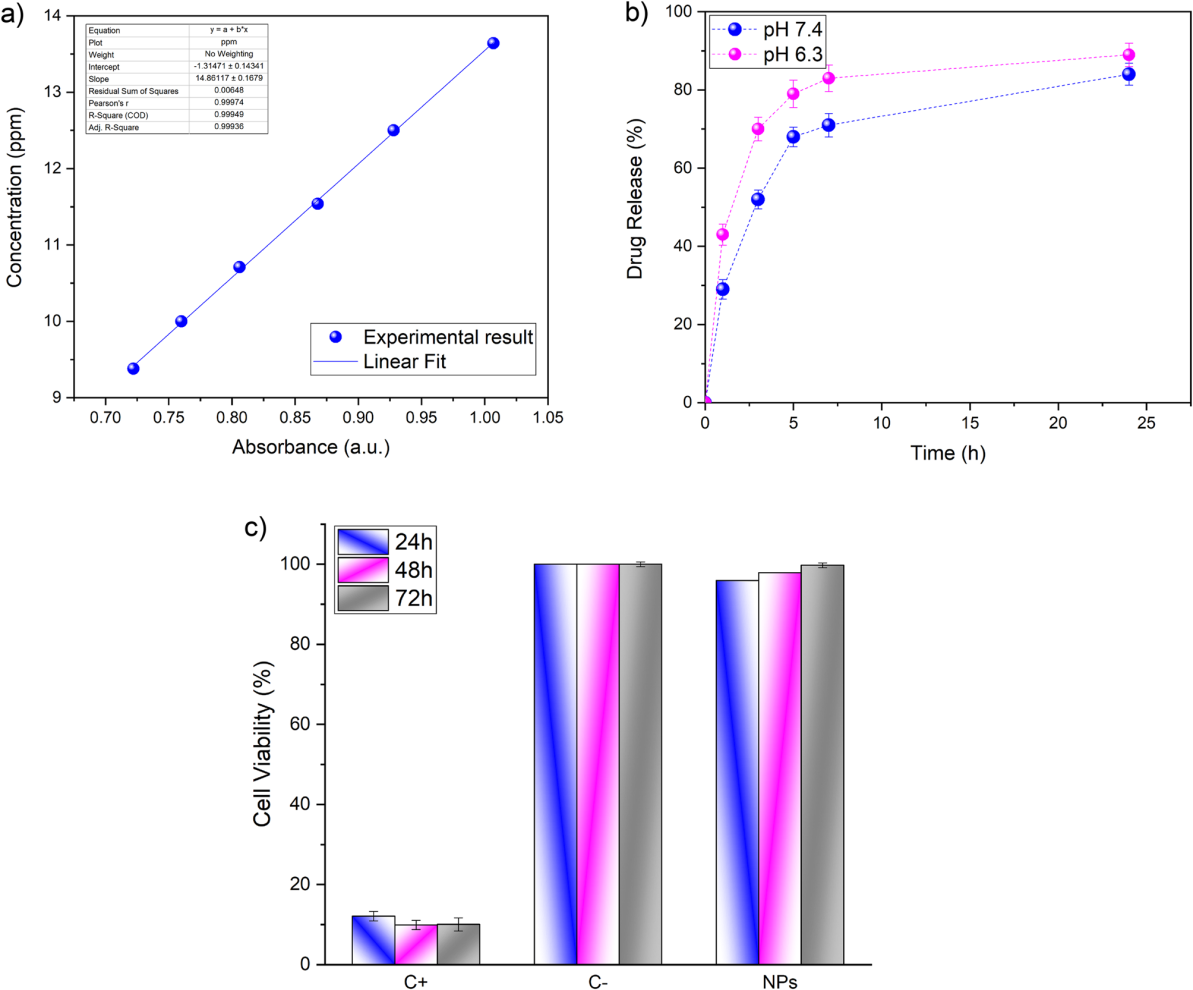
In vitro studies; (**a**) Standard calibration curve of PHT; (**b**) The DR profile at different pH from PHT-loaded IO@Si@Gel MNPs; (**c**) MTT assay of the IO@Si@Gel MNPs treated on L-929 cells.

To gather reliable data regarding the PHT release characteristics of NPs, it is essential to evaluate the release properties of the MNPs within biological fluids. Hence, in vitro release studies in physiological conditions and acidic cancerous environments were studied for up to 24 h. Firstly, the standard calibration curve of PHT was plotted (Fig. 6a) and the calibration $y = 14.8611x - 1.3147, R^{2} = 0.9994$ Eq. 4 of the drug was obtained:

4 $$y = 14.8611x - 1.3147, R^{2} = 0.9994$$

It has been reported that the pH value in the brain tumor region is 6.8, compared to healthy brain tissue and blood fluid (with a pH value of 7.4)^63^. Moreover, during seizures, the intracellular pH value decreases by a mean of 0.5 units^10^. Hence, the in vitro release of PHT was done in PBS (pH 7.4) and the simulated tumorous-epileptic pH condition (pH 6.3) was at 37 °C to mimic the blood and cancerous-epileptic environments, respectively. The DR profile findings reveal an initial fast release of PHT from the NPs, amounting to 68 and 79%, within the initial 5 h, and a similar observation was reported by Kazmi et al.^46^, albeit for a different pH-responsive system. Subsequently, a more gradual and sustained release is observed, culminating in 84 and 89% release at pH 7.4 and pH 6.3, respectively, over a 24-h period, respectively (Fig. 6b). The highest DR values in lower pH indicated that these NPs are pH-responsive, and this system can increase the DR rate in a tumorous and epileptic environment, where the pH value is in a range of 6–7.

To assess PHT release, various kinetic models, i.e. the Parabolic Diffusion, Higuchi, zero-order, and first-order models were employed to determine the release constant and R^2^ values, as summarized in Table 2. Among these models, the Parabolic Diffusion Model demonstrated the best fit for characterizing the mechanism of PHT release from PHT-loaded IO@Si@Gel MNPs, as indicated by higher R^2^ values in both pH conditions. The Korsmeyer-Peppas equation was utilized to fit the data as follows:

5 $$Mt/Me = kt^{n}$$

**Table 2:** The correlation coefficient (R^2^) determined from several models fitted to the DR data under two pH values.

pH value	Zero-order ${\text{C}}_{{\text{t}}} = {\text{ C}}_{0} + {\text{ K}}_{0} {\text{t}}$	First-order ${\text{dC}}/{\text{dt}} = \, - {\text{K}}_{{1}} {\text{t}}$	Higuchi ${\text{Q}} = {\text{K}}_{{\text{H}}} {\text{t}}^{0.5}$	Korsmeyer peppas ${\text{M}}_{{\text{t}}} /{\text{M}}_{{\text{e}}} = {\text{Kt}}^{{\text{n}}}$	Parabolic diffusion $(1 - {\text{Ct}}/{\text{C0}})/{\text{t}} = {\text{Kt}}^{{ - 0.5 + {\text{a}}}}$
7.4	y = 2.5712x + 33.525 R^2^ = 0.5347	y = − 0.0061x + 1.1231 R^2^ = 0.024	y = 17.008x + 15.196 R^2^ = 0.8271	y = 0.3356x + 1.529 R^2^ = 0.8778 , n = 0.33	y = 0.0281x R^2^ = 0.9347
6.3	y = 2.3856x + 44.763 R^2^ = 0.3915	y = − 0.0283x + 1.3059 R^2^ = 0.3547	y = 17.072x + 25.063 R^2^ = 0.7089	y = 0.2253x + 1.6957 R^2^ = 0.8124 , n = 0.22	y = 0.0322x R^2^ = 0.893

In the equation, *Me* is the amount of equilibrium drug released at, while *Mt* is the amount of drug released at time *t*. The parameters* k* and *n* correspond to a characteristic constant and a characteristic exponent, respectively, which are associated with the transport mechanism (diffusional exponent). The n values of PHT-loaded IO@Si@Gel MNPs were 0.33 and 0.22 at pH 7.4 and pH 6.3, respectively, which showed that the release process follows Fickian diffusion^47^.

The uncontrolled drug administration approach causes insufficient amounts of cellular drug uptake or extreme toxic levels due to overdose, whereas the smart drug delivery system with stimuli-responsive properties controls and improves the release of drugs for a prolonged time and with increased therapeutic efficacy^64^. This system is categorized into two types including self-regulated and externally stimuli-regulated mechanisms. The self-regulated mechanism adapts the DR rate by detecting variations in pH, concentration, or temperature. Conversely, externally stimuli-regulated systems sustain DR through external stimuli like a MF or ultrasound waves^65^. In the current study, gelatin, as a smart polymer, displays a self-regulated release, in response to pH changes, while the magnetic core part of the NPs, as an externally addressable part, could be used for directing the delivery to the specific region. In the case of externally stimuli-regulated mechanisms, it has been demonstrated that microbubbles formed by ultrasound can open the tight junctions of BBB and help the medications enter CNS disorders^65^. In addition, non-invasive focused ultrasound therapy causes a reduction in the progression of epilepsy^65^. Hence, the future direction of research in the field of smart multifunctional NPs for brain disorders with epileptic symptoms could be developing a novel technique for deeper penetration effects across the BBB (such as ultrasound-responsive neuromodulation) and facilitating the translation of the smart NPs from bench to clinic.

To assess the biocompatibility and the cell viability effect of the synthesized NPs, a normal L929 cell line was employed and an MTT assay was adopted based on the live-cell mitochondria reactions. In this study, the L-929 cells were incubated with 10 mg ml^−1^ of the IO@Si@Gel MNPs, while the L-929 cells, without any treatment, were selected as the negative control group (C −). In addition, the positive control group (C +) contained L-929 cells with Latex extracted in the culture medium with 10% FBS. As observed by the MTT assay (Fig. 6c), 95.9% of cell viability was detected when they were cultured with the NPs after 1 day, indicating the biosafety of the nanocarriers. In the case of 48 h, when L-929 cells were cultured with the NPs, the cell viability reached 97.9%, confirming no direct cytotoxic effects. The percentage of cell viability increased to 99.8 after 72 h. These results showed that the IO@Si@Gel MNPs can be applied as biocompatible agents and possess no cytotoxicity.

## Conclusions

The smart and multifunctional superparamagnetic NPs with a core-double shell structure were synthesized in this study. These were found to be capable of drug delivery to a simulated tumorous-epileptic microenvironment. Comprehensive tests confirmed that the superparamagnetic NP cores were enveloped by a layer of silica and a layer of Gel, resulting in NPs with an overall dimension of 41 nm. This falls within the targeted size range for facilitating BBB transmigration. Additionally, the specific saturation magnetization value of the ultimate MNPs was measured at emu g^−1^. The VSM results showed that despite coating, the inherent magnetic property was retained and served final NPs as good MRI contrast agents. The polymeric shell could load the anti-epileptic drug with a DL capacity and EE of 2.01 and 10.05%, respectively. It was shown that this layer released more amount of drugs in an acidic condition in comparison with a natural condition, indicating a pH-sensitive system. In vitro studies revealed a Fickian diffusion process for PHT release (as diffusional exponent values based on the Korsmeyer-Peppas equation were obtained as 0.33 and 0.22 at pH 7.4 and pH 6.3, respectively) and a biocompatible behavior for synthesized NPs. The developed NP capability to be guided by an external MF gradient to the desired site makes them a good candidate for brain disorders’ theranostics applications.

## Data Availability

The datasets generated during and/or analyzed during the current study are available from the corresponding author upon reasonable request.

## References

[1] Kim S (2023). Risk of epilepsy in gonadal teratoma: A nationwide population-based study. Sci. Rep..

[2] Huntoon K, Musgrave N, Shaikhouni A, Elder J (2023). Frequency of seizures in patients with metastatic brain tumors. Neurol. Sci..

[3] Kermanshahi N (2024). The Prevalence of seizures in brain metastasis patients on anticonvulsant prophylaxis: A systematic review and meta-analysis. World Neurosurg..

[4] Beaumont A, Whittle I (2000). The pathogenesis of tumour associated epilepsy. Acta Neurochir..

[5] Siomin V, Angelov L, Li L, Vogelbaum MA (2005). Results of a survey of neurosurgical practice patterns regarding the prophylactic use of anti-epilepsy drugs in patients with brain tumors. J. Neurooncol..

[6] Shekaari H, Zafarani-Moattar MT, Mokhtarpour M, Faraji S (2021). Deep eutectic solvents for antiepileptic drug phenytoin solubilization: Thermodynamic study. Sci. Rep..

[7] Keppel Hesselink JM, Kopsky DJ (2017). Phenytoin: 80 years young, from epilepsy to breast cancer, a remarkable molecule with multiple modes of action. J. Neurol..

[8] Kim E (2023). Transcranial focused ultrasound-mediated unbinding of phenytoin from plasma proteins for suppression of chronic temporal lobe epilepsy in a rodent model. Sci. Rep..

[9] Patsalos PN, Spencer EP, Berry DJ (2018). Therapeutic drug monitoring of antiepileptic drugs in epilepsy: A 2018 update. Ther. Drug Monit..

[10] Siesjö BK, von Hanwehr R, Nergelius G, Nevander G, Ingvar M (1985). Extra-and intracellular pH in the brain during seizures and in the recovery period following the arrest of seizure activity. J. Cereb. Blood Flow Metab..

[11] Janib SM, Moses AS, MacKay JA (2010). Imaging and drug delivery using theranostic nanoparticles. Adv. Drug Deliv. Rev..

[12] Saha N (2023). Advanced radio frequency applicators for thermal magnetic resonance theranostics of brain tumors. Cancers.

[13] Sharma R, Das O, Damle SG, Sharma AK (2013). Isocitrate lyase: A potential target for anti-tubercular drugs. Recent Pat. Inflamm. Allergy Drug Discov..

[14] Sharma AK, Kumar R, Nishal B, Das O (2013). nanocarriers as promising drug vehicles for the management of tuberculosis. BioNanoScience.

[15] Yerpude ST (2023). Biomedical, clinical and environmental applications of platinum-based nanohybrids: An updated review. Environ. Res..

[16] Serajian A, Hassanpour M, Heidari G (2023). Drug delivery for brains and central nervous system. Mater. Chem. Horizons.

[17] Rabiee N, Iravani S (2023). MXenes and their composites: A versatile platform for biomedical applications. Mater. Chem. Horizons.

[18] Kaur S (2018). Bioengineered PLGA-chitosan nanoparticles for brain targeted intranasal delivery of antiepileptic TRH analogues. Chem. Eng. J..

[19] Wilson B, Lavanya Y, Priyadarshini S, Ramasamy M, Jenita JL (2014). Albumin nanoparticles for the delivery of gabapentin: Preparation, characterization and pharmacodynamic studies. Int. J. Pharm..

[20] Argyo C, Weiss V, Bräuchle C, Bein T (2014). Multifunctional mesoporous silica nanoparticles as a universal platform for drug delivery. Chem. Mater..

[21] Wei H (2017). Exceedingly small iron oxide nanoparticles as positive MRI contrast agents. Proc. Natl. Acad. Sci..

[22] Su X (2017). A graphene quantum dot@ Fe3O4@ SiO2 based nanoprobe for drug delivery sensing and dual-modal fluorescence and MRI imaging in cancer cells. Biosens. Bioelectron..

[23] Schaller B (2005). Influences of brain tumor-associated pH changes and hypoxia on epileptogenesis. Acta Neurol. Scand..

[24] de Oliveira EG (2018). Reconstituted spray-dried phenytoin-loaded nanocapsules improve the in vivo phenytoin anticonvulsant effect and the survival time in mice. Int. J. Pharm..

[25] Wang Y (2016). Electroresponsive nanoparticles improve antiseizure effect of phenytoin in generalized tonic-clonic seizures. Neurotherapeutics.

[26] Rosillo-de la Torre A (2015). Phenytoin carried by silica core iron oxide nanoparticles reduces the expression of pharmacoresistant seizures in rats. Nanomedicine.

[27] Liu S, Yang S, Ho PC (2018). Intranasal administration of carbamazepine-loaded carboxymethyl chitosan nanoparticles for drug delivery to the brain. Asian J. Pharm. Sci..

[28] López T, Cuevas J, Jardón G, Gómez E, Ramirez P (2015). Preparation and characterization of antiepileptic drugs encapsulated in sol-gel titania nanoparticles as controlled release system. Med. Chem..

[29] Motawea A, Borg T, Abd El-Gawad AE (2018). Topical phenytoin nanostructured lipid carriers: Design and development. Drug Dev. Ind. Pharm..

[30] Senthilvel C, Karuppaiyan K, Moideen M (2019). Development of capsules filled with phenytoin and berberine loaded nanoparticles—a new approach to improve anticonvulsant efficacy. Indian J. Pharm. Educ. Res.

[31] Zhao J (2020). Nanocage encapsulation improves antiepileptic efficiency of phenytoin. Biomaterials.

[32] de Oliveira EG (2020). Phenytoin-loaded lipid-core nanocapsules improve the technological properties and in vivo performance of fluidised bed granules. Mater. Sci. Eng.: C.

[33] Kumar S, Madhav NVS, Verma A, Pathak K (2020). A smart approach for delivery of nanosized phenytoin using biomaterial isolated from Fragaria ananassa. Int. J. Pharm. Investig..

[34] Yousfan A (2020). Preparation and characterisation of PHT-loaded chitosan lecithin nanoparticles for intranasal drug delivery to the brain. RSC Adv..

[35] Nair SC, Vinayan KP, Mangalathillam S (2021). Nose to brain delivery of phenytoin sodium loaded nano lipid carriers: Formulation, drug release, permeation and in vivo pharmacokinetic studies. Pharmaceutics.

[36] Wu D (2022). Nanoengineered on-demand drug delivery system improves efficacy of pharmacotherapy for epilepsy. Sci. Adv..

[37] Suneetha SCA, Raghupathy BPC, Suresh P (2014). Physicochemical characterization and cytotoxicity screening of a novel colloidal nanogold-based phenytoin conjugate. Sci. Pharm..

[38] Yin L, Hillmyer MA (2014). Preparation and performance of hydroxypropyl methylcellulose esters of substituted succinates for in vitro supersaturation of a crystalline hydrophobic drug. Mol. Pharm..

[39] Oh JK, Park JM (2011). Iron oxide-based superparamagnetic polymeric nanomaterials: Design, preparation, and biomedical application. Progr. Polym. Sci..

[40] Glantz M (2000). Practice parameter: Anticonvulsant prophylaxis in patients with newly diagnosed brain tumors. Neurology.

[41] Ghane N, Mazinani S, Gharehaghaji AA (2018). Fabrication and characterization of hollow nanofibrous PA6 yarn reinforced with CNTs. J. Polym. Res..

[42] Ghane N, Mazinani S, Gharehaghaji A (2019). Comparing the performance of electrospun and cast nanocomposite film of polyamide-6 reinforced with multi-wall carbon nanotubes. J. Plast. Film Sh..

[43] Ghane N (2021). Regeneration of the peripheral nerve via multifunctional electrospun scaffolds. J. Biomed. Mater. Res. Part A.

[44] Ghane N, Beigi M-H, Labbaf S, Nasr-Esfahani M-H, Kiani A (2020). Design of hydrogel-based scaffolds for the treatment of spinal cord injuries. J. Mater. Chem. B.

[45] Zou Z (2013). Natural gelatin capped mesoporous silica nanoparticles for intracellular acid-triggered drug delivery. Langmuir.

[46] Kazmi SAR, Qureshi MZ, Ali S, Masson J-F (2019). In vitro drug release and biocatalysis from pH-responsive gold nanoparticles synthesized using doxycycline. Langmuir.

[47] Khalili S (2022). Cytocompatibility and antibacterial properties of coaxial electrospun nanofibers containing ciprofloxacin and indomethacin drugs. Polymers.

[48] Mahmoudi M, Sant S, Wang B, Laurent S, Sen T (2011). Superparamagnetic iron oxide nanoparticles (SPIONs): Development, surface modification and applications in chemotherapy. Adv. Drug Deliv. Rev..

[49] Chomoucka J (2010). Magnetic nanoparticles and targeted drug delivering. Pharmacol. Res..

[50] Roushani M, Saraei S, Zare Dizajdizi B, Valipour A (2021). Preparation of magnetic imprinted polymer nanoparticle carbon paste electrode for determination of valproic acid. Adv. Nanochem..

[51] Sun K (2018). Enhanced highly toxic reactive oxygen species levels from iron oxide core–shell mesoporous silica nanocarrier-mediated Fenton reactions for cancer therapy. J. Mater. Chem. B.

[52] Sun L (2010). Synthesis of magnetic and fluorescent multifunctional hollow silica nanocomposites for live cell imaging. J. Colloid Interface Sci..

[53] Zhao H, Lu H, Gong T, Zhang Z (2013). Nanoemulsion loaded with lycobetaine–oleic acid ionic complex: Physicochemical characteristics, in vitro, in vivo evaluation, and antitumor activity. Int. J. Nanomed..

[54] Sneha M, Sundaram NM (2015). Preparation and characterization of an iron oxide-hydroxyapatite nanocomposite for potential bone cancer therapy. Int. J. Nanomed..

[55] Hauptman JS, Safaee M (2010). From the bench to the bedside: Spinal cord regeneration, niacin for stroke, magnetic nanoparticles, stimulation for epilepsy, role of galanins in epilepsy, functions of the supramarginal gyri, and the role of inflammation in postoperative cognitive disturbances. Surg. Neurol. Int..

[56] Sanganeria P (2015). Cellular internalization and detailed toxicity analysis of protein-immobilized iron oxide nanoparticles. J. Biomed. Mater. Res. Part B: Appl. Biomater..

[57] Maier-Hauff K (2011). Efficacy and safety of intratumoral thermotherapy using magnetic iron-oxide nanoparticles combined with external beam radiotherapy on patients with recurrent glioblastoma multiforme. J. Neuro-oncol..

[58] Tan J (2019). I6P7 peptide modified superparamagnetic iron oxide nanoparticles for magnetic resonance imaging detection of low-grade brain gliomas. J. Mater. Chem. B.

[59] Ebadi M (2021). Drug delivery system based on magnetic iron oxide nanoparticles coated with (polyvinyl alcohol-zinc/aluminium-layered double hydroxide-sorafenib). Alex. Eng. J..

[60] Gupta AK, Gupta M (2005). Synthesis and surface engineering of iron oxide nanoparticles for biomedical applications. Biomaterials.

[61] Tadmor R, Rosensweig RE, Frey J, Klein J (2000). Resolving the puzzle of ferrofluid dispersants. Langmuir.

[62] Kumar CS, Mohammad F (2011). Magnetic nanomaterials for hyperthermia-based therapy and controlled drug delivery. Adv. Drug Deliv. Rev..

[63] Rao JU (2017). Temozolomide arrests glioma growth and normalizes intratumoral extracellular pH. Sci. Rep..

[64] Das SS (2020). In Stimuli responsive polymeric nanocarriers for drug delivery applications. Polymers.

[65] Shende P, Trivedi R (2021). Nanotheranostics in epilepsy: A perspective for multimodal diagnosis and strategic management. Nano Select.

